# VelcroVax: a “Bolt-On” Vaccine Platform for Glycoprotein Display

**DOI:** 10.1128/msphere.00568-22

**Published:** 2023-01-31

**Authors:** Natalie J. Kingston, Keith Grehan, Joseph S. Snowden, Mark Hassall, Jehad Alzahrani, Guido C. Paesen, Lee Sherry, Connor Hayward, Amy Roe, Sam Stephen, Darren Tomlinson, Antra Zeltina, Katie J. Doores, Neil A. Ranson, Martin Stacey, Mark Page, Nicola J. Rose, Thomas A. Bowden, David J. Rowlands, Nicola J. Stonehouse

**Affiliations:** a Astbury Centre for Structural Molecular Biology, School of Molecular and Cellular Biology, Faculty of Biological Sciences, University of Leeds, Leeds, United Kingdom; b Division of Virology, National Institute for Biological Standards and Control (NIBSC), Hertfordshire, United Kingdom; c Division of Structural Biology, Wellcome Centre for Human Genetics, University of Oxford, Oxford, United Kingdom; d Department of Infectious Diseases, School of Immunology and Microbial Sciences, King's College London, United Kingdom; University of Maryland School of Medicine

**Keywords:** HBcAg, VLP, Junín virus, platform, vaccine

## Abstract

Having varied approaches to the design and manufacture of vaccines is critical in being able to respond to worldwide needs and newly emerging pathogens. Virus-like particles (VLPs) form the basis of two of the most successful licensed vaccines (against hepatitis B virus [HBV] and human papillomavirus). They are produced by recombinant expression of viral structural proteins, which assemble into immunogenic nanoparticles. VLPs can be modified to present unrelated antigens, and here we describe a universal “bolt-on” platform (termed VelcroVax) where the capturing VLP and the target antigen are produced separately. We utilize a modified HBV core (HBcAg) VLP with surface expression of a high-affinity binding sequence (Affimer) directed against a SUMO tag and use this to capture SUMO-tagged gp1 glycoprotein from the arenavirus Junín virus (JUNV). Using this model system, we have solved the first high-resolution structures of VelcroVax VLPs and shown that the VelcroVax-JUNV gp1 complex induces superior humoral immune responses compared to the noncomplexed viral protein. We propose that this system could be modified to present a range of antigens and therefore form the foundation of future rapid-response vaccination strategies.

**IMPORTANCE** The hepatitis B core protein (HBc) forms noninfectious virus-like particles, which can be modified to present a capturing molecule, allowing suitably tagged antigens to be bound on their surface. This system can be adapted and provides the foundation for a universal “bolt-on” vaccine platform (termed VelcroVax) that can be easily and rapidly modified to generate nanoparticle vaccine candidates.

## INTRODUCTION

The need for safe and effective vaccines to be developed rapidly and distributed globally has been highlighted over the last 2 years. Vaccines have been developed for more than 20 different pathogens, and more than 15 additional organisms are recognized by the World Health Organization (WHO) as priority pathogens with epidemic or pandemic potential. Although the WHO endeavors to accelerate the development of vaccines for these priority pathogens for use in low- and middle-income countries (LMICs), there are significant challenges to their development and deployment (1). These include safety, efficacy, and the need to maintain a cold chain when delivering vaccines to remote areas. Importantly, the availability of vaccines in regions of endemicity is essential to control the spread of pathogens and facilitate the prevention of future global pandemics.

The list of pathogens with epidemic or pandemic potential varies among global authorities. The National Institute of Allergy and Infectious Diseases (NIAID) priority list includes some new world arenaviruses, including Junín virus (JUNV), which causes a potentially lethal hemorrhagic disease known as Argentine hemorrhagic fever (AHF) (2, 3). JUNV is transmitted via rodents (Calomys musculinus) and contracted via contact with infected excretions or aerosols. Outbreaks of AHF in the 1960s and 1970s resulted in thousands of deaths and had case fatality rates between 15 and 30% (4–6). Total cases decreased in the following decades and have fallen substantially since the introduction of a live attenuated vaccine in affected regions of Argentina (6–8). Despite the success of this vaccine, as with all attenuated virus vaccines, there remain safety concerns regarding the potential for reversion to a pathogenic form.

The advancement of technologies used for vaccine production and purification has contributed to the generation of safer vaccines. For example, virus-like particle (VLP) vaccines for hepatitis B virus (HBV) and human papillomavirus (HPV) have shown exemplary safety and efficacy (9, 10). Most recently, recombinant nonreplicating viral vectors and RNA vaccines have been produced rapidly and also show impressive safety and efficacy profiles (11–13). Critically, in contrast to attenuated vaccines, inactivated, subunit, polysaccharide, RNA, or toxoid vaccines are nonreplicative, so they do not pose the risk of reversion to a pathogenic form. This makes recombinant technologies the most attractive approach for the development of safer next-generation vaccines.

The efficacy of subunit vaccines can be enhanced when the subunit exists as a nanoparticle. Nanoparticles may be naturally occurring (VLPs), artificially formed (14–16), or modified biological chimeras (17–19). Indeed, chimeric VLP technology has allowed the deployment of the first licensed anti-malaria vaccine, Mosquirix (18, 20). The success of this vaccine suggests that a chimeric VLP approach is both tractable and suitable for improving responses against challenging immunogens; however, it took over 30 years for Mosquirix to be licensed (18, 21). Alternative approaches for modifying VLPs have been investigated to increase the diversity of vaccine platforms. The approach we have pursued relies on the surface display of a capturing molecule (e.g. antibody, nanobody) on a nanoparticle carrier, which is able to bind and display an antigen of interest tagged with an appropriate sequence. Poorly immunogenic antigens displayed on nanoparticles are more effectively recognized by dendritic cells (DCs). In addition, nanoparticle size (30 to 100 nm) can influence T-helper (Th) bias and Th epitopes from protein-based nanoparticles can contribute to immunity; thus, humoral responses generated are likely to be a higher affinity and more diverse (22–26).

Here, we describe a vaccine system in which a carrier nanoparticle and antigen are produced separately. We have developed a modified HBV core (HBcAg) VLP, termed VelcroVax, with surface expression of a SUMO-Affimer. Affimers are produced by phage display approaches, are small (~13 kDa), and can be expressed in a range of systems (27). We used these VLPs to capture the SUMO-tagged JUNV glycoprotein, gp1. In common with the wild-type HBcAg protein, the modified VelcroVax protein assembles as both *T *= 3 or *T *= 4 particles, composed of 90 or 120 core protein dimers, respectively. Using this model system, we characterize VelcroVax structurally and functionally, using comparative immunization trials to show that JUNV gp1 coupling to VLPs alters the immune response generated both quantitatively and qualitatively. We propose that this system may be modified for a range of antigens in the future and could form the foundation of future rapid-response vaccination strategies.

## RESULTS

### Generation of VelcroVax.

**FIG 1:**
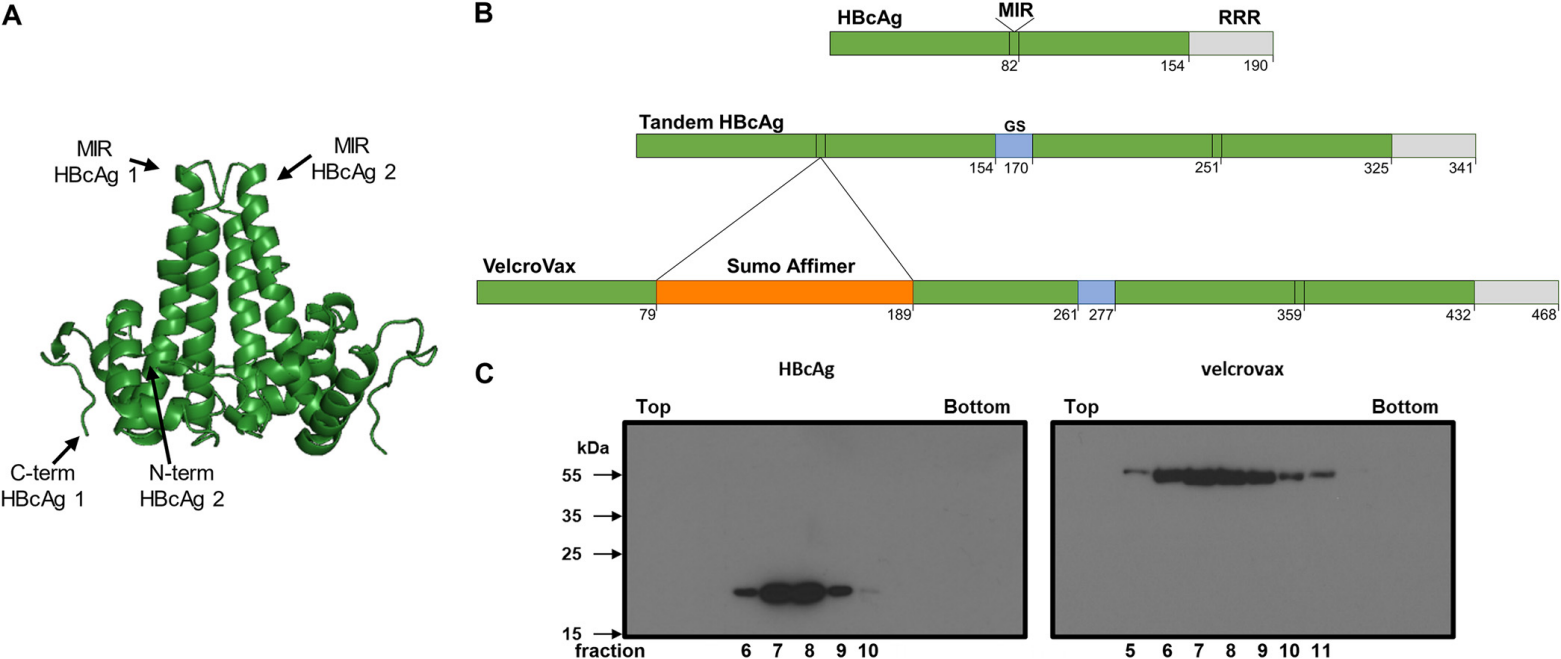
Generation of HBcAg and VelcroVax in Pichia pastoris. (A) X-ray crystal structure of a HBcAg dimer (PDB: 1QGT; reference 29). The locations of the C-terminal end of monomer 1 and N-terminal end of monomer 2 and the major immunodominant regions (MIR) are indicated. (B) Organization of HBcAg, tandem HBcAg, and VelcroVax constructs with amino acid positions indicated. Representation depicts MIR, arginine-rich repeat (RRR), glycine-serine linking sequence (GS) and the insertion site of a SUMO Affimer within the MIR of the first of the fused HBcAg monomers. (C) Anti-HBcAg Western blot of gradient purified HBcAg and VelcroVax particles produced in Pichia pastoris, probed with 10E11, is shown; *n* = 3.

HBcAg monomers assemble into paired dimers, which further assemble to form *T *= 3 (90 dimers) and *T *= 4 (120 dimers) symmetric particles. Within each dimer, the C-terminal end of one monomer is in proximity to the N-terminal end of the other partner (Fig. 1A) (28, 29). The genetic fusion of these monomers using a sequence encoding a Gly-Ser linker ensures that the genetically fused pairs will dimerize within the assembled VLP, termed tandem HBcAg (tHBcAg). Unlike previous studies which utilized the second HBcAg monomer of the major immunodominant region (MIR) as the insertion site (28), we elected to insert a sequence encoding a SUMO-Affimer into the first HBcAg monomer within the tandem construct (Fig. 1B). This organization ensures that within each HBcAg dimer, one MIR will contain a SUMO-Affimer and the other will not, functionally minimizing the likelihood of steric clashes within this region. This construct, with the SUMO-Affimer within the MIR of the first HBcAg monomer within a fused dimer, is the first example of the VelcroVax system.

To determine whether the introduction of a SUMO-Affimer sequence within the MIR of a tandem HBcAg construct was compatible with particle formation, we used Pichia pastoris as an expression system. Samples of wild-type (wt) HBcAg or VelcroVax were produced in P. pastoris and separated through a 15 to 45% sucrose gradient (Fig. 1C). Western blot analysis using anti-HBcAg antibody 10E11 indicated that both wt HBcAg and VelcroVax particles were present within the gradient. For both particle types, the signal peaked around fraction 8, indicating that both wt HBcAg and VelcroVax effectively form VLPs in this system. The presence of particles corresponding to the sizes of both *T *= 3 and *T *= 4 VLPs was confirmed by negative stain electron microscopy (EM) (Fig. S1).

10.1128/msphere.00568-22.1 FIG S1 Characterization of unmodified HBcAg and VelcroVax by negative stain EM. Representative micrographs of unmodified HBcAg (WT) and VelcroVax. For each, scale bars represent 200 nm (full micrograph) or 50 nm (expanded inset). Download FIG S1, TIF file, 0.4 MB. Copyright © 2023 Kingston et al. 2023 Kingston et al. https://creativecommons.org/licenses/by/4.0/ This content is distributed under the terms of the Creative Commons Attribution 4.0 International license.

### Structural characterization of VelcroVax.

**FIG 2:**
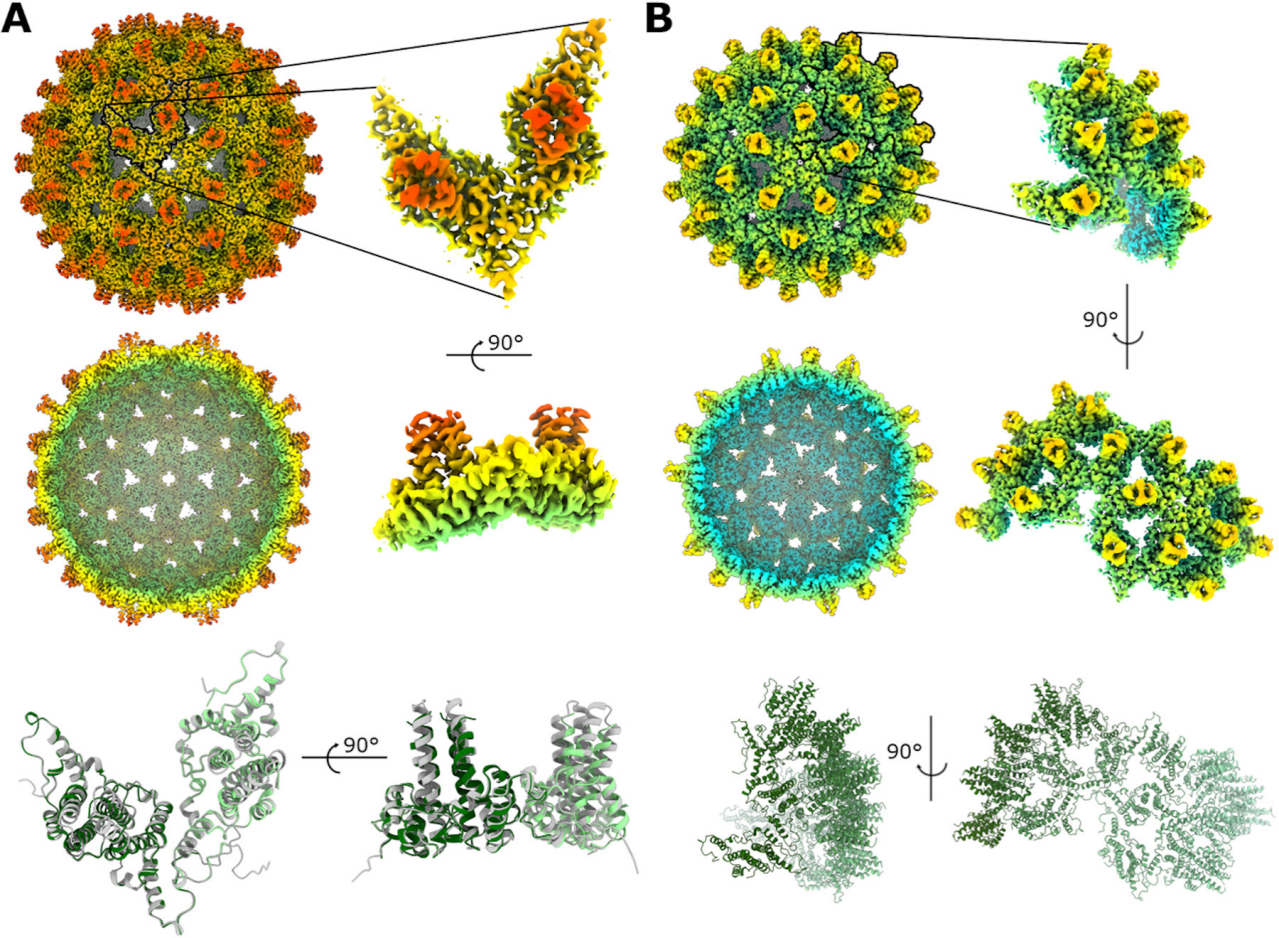
Structural characterization of VelcroVax VLPs. (A and B) Full and sectional isosurface representations of density maps for *T *= 4 (A) and *T *= 3* (B) VelcroVax VLPs, filtered by local resolution, shown at the same contour level and colored according to the same radial coloring scheme. In each case, an expanded view of an individual asymmetric unit (*T *= 4–I1 symmetry; *T *= 3*–C5 symmetry) and corresponding atomic models are shown. For the *T *= 4 asymmetric unit, the VelcroVax atomic model (green) is overlaid with the cryoEM structure of wt HBcAg (gray, PDB: 7OD4; reference 30).

To characterize VelcroVax structurally and assess the impact of SUMO-Affimer insertion, we generated high-resolution structures of VelcroVax VLPs. Notably, as a result of the tandem arrangement of VelcroVax, the *T *= 3 configuration does not conform to icosahedral symmetry. Each VelcroVax subunit comprises two connected HBcAg monomers, only one of which is modified with an Affimer, generating an imbalance between what would be true icosahedral asymmetric units (Fig. S3A). As such, this configuration was termed *T *= 3* rather than *T *= 3, and 5-fold (C5) symmetry was imposed during image processing rather than icosahedral symmetry (I1), which was imposed for the *T *= 4 VLP. Freshly purified VLPs were used for cryoEM data collection, and structures were determined for both *T *= 4 (at 2.9-Å resolution) and *T *= 3* (at 3.6-Å resolution) configurations (Fig. 2; Fig. S2).

10.1128/msphere.00568-22.2 FIG S2 VelcroVax cryoEM data collection and image processing. (A) Representative micrograph from VelcroVax cryoEM dataset. Scale bar indicates 100 nm. (B) Representative class averages from 2D classification of VelcroVax particles, including both *T *= 4 and *T *= 3* VLPs. These were used in the early steps of analysis to generate initial models. (C) Fourier shell correlation (FSC) plots for final reconstructions of *T *= 4 (left) and *T *= 3* (right) VLPs. Nominal resolutions are indicated and were determined using the FSC = 0.143 criterion with high-resolution noise substitution to correct for any overfitting (black line, “corrected”). Download FIG S2, TIF file, 0.6 MB. Copyright © 2023 Kingston et al. 2023 Kingston et al. https://creativecommons.org/licenses/by/4.0/ This content is distributed under the terms of the Creative Commons Attribution 4.0 International license.

10.1128/msphere.00568-22.3 FIG S3 Inherent asymmetry within VelcroVax subunits. (A) Schematic illustrating how the tandem nature of VelcroVax does not conform to icosahedral symmetry in the *T *= 3* arrangement. Each VelcroVax monomer is formed from a tandem HBc subunit (green) linked by a flexible linker (beige) and a single Affimer (orange). This does not fit within the strict asymmetric unit (pink) of a true *T *= 3 VLP. (B) VelcroVax subunits can be incorporated into the asymmetric unit (here, *T *= 4) in either direction, leading to variation in the position of the Affimers. This results in blurring of Affimer density when particles are averaged to generate cryoEM reconstructions of VelcroVax VLPs. Download FIG S3, TIF file, 0.5 MB. Copyright © 2023 Kingston et al. 2023 Kingston et al. https://creativecommons.org/licenses/by/4.0/ This content is distributed under the terms of the Creative Commons Attribution 4.0 International license.

In general, VelcroVax showed a high level of structural similarity to unmodified HBcAg. For comparison, the atomic model for *T *= 4 VelcroVax was aligned with the best-matched subunit from a 2.8 Å resolution cryoEM structure of a *T *= 4 HBcAg VLP (PDB: 7OD4; reference 30). A root mean square deviation (RMSD) value calculated between equivalent Cα atoms was only ~1.5 Å, and visual inspection revealed a high degree of overlap (Fig. 2A). Most of the variation appeared to localize to the four-helix bundles, as might be expected given the proximity of this region to the inserted Affimer in VelcroVax.

**FIG 3:**
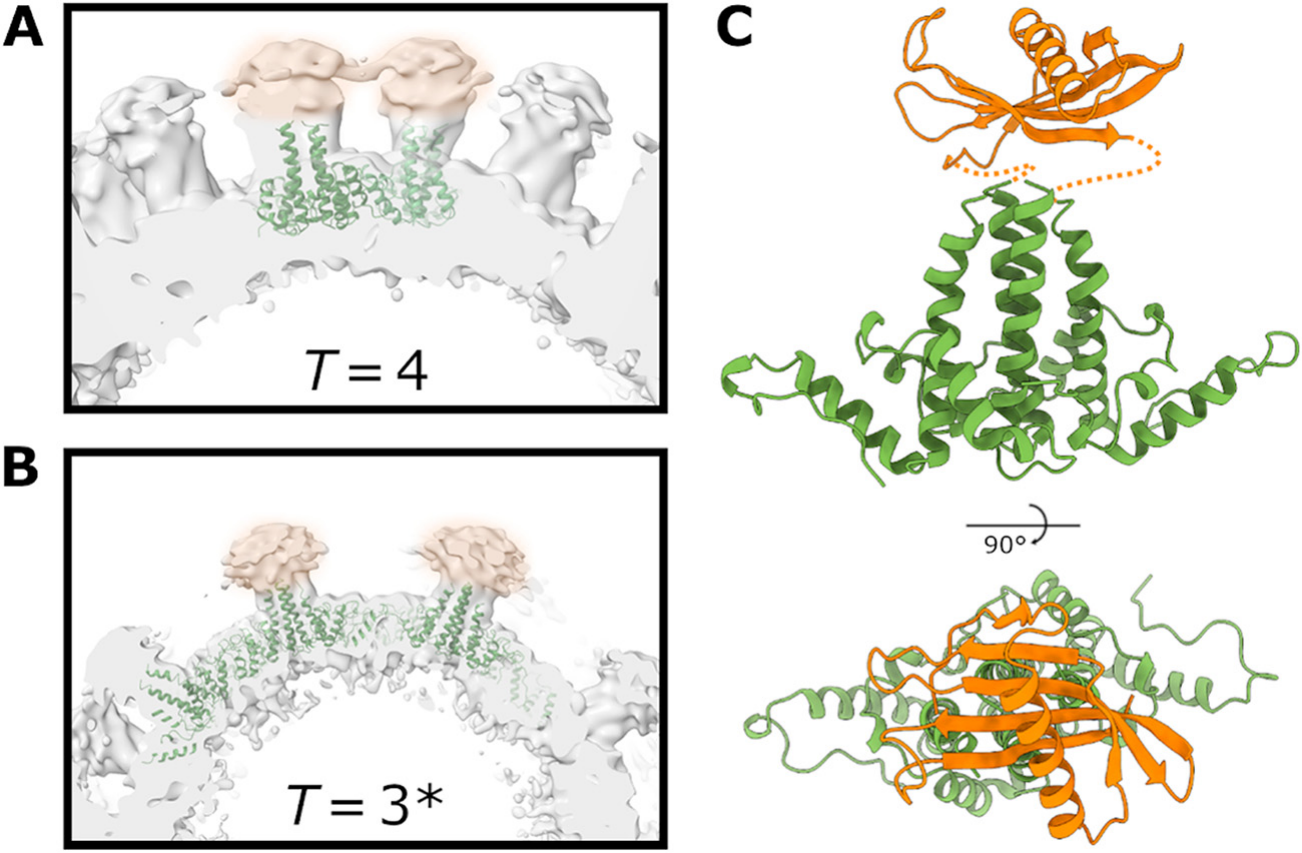
Affimer density in low-pass filtered VelcroVax VLP reconstructions. (A and B) Sections of local resolution-filtered density maps for (A) *T *= 4 and (B) *T *= 3* VelcroVax VLPs following application of a 10-Å low-pass filter. Amorphous Affimer density (orange highlight) is visible above VelcroVax four-helix bundles. (C) Atomic model for a single VelcroVax monomer (green) with a SUMO-Affimer homology model (orange) manually positioned above the four-helix bundle, indicating the expected position of the Affimer based on the density shown in panels A and B.

Although the majority of the VLP was well resolved, density for the SUMO-Affimer was not evident in reconstructions of either *T = *4 or *T *= 3* VelcroVax VLPs. However, at low contour levels, weak, diffuse density was visible above four-helix bundles. For both *T *= 4 and *T *= 3* VLPs, maps low-pass filtered to 10 Å revealed additional density above the four-helix bundles consistent with the expected size of the Affimer (Fig. 3), confirming that Affimers were likely present but were not resolved to high resolution.

In an attempt to resolve Affimer density, data for the *T *= 4 configuration of VelcroVax was subjected to symmetry expansion and focused 3D classification, using a mask to isolate the region above the four-helix bundle. However, while there was considerable variation between classes, none of the classes contained well-resolved Affimer density (Fig. S4), confirming the high level of variability in Affimer positioning. Because of its unique symmetrical properties and therefore much more limited chance of success, focused classification was not attempted for *T *= 3* data.

10.1128/msphere.00568-22.4 FIG S4 Focused classification failed to resolve Affimer density. (A) Focused classification was performed with a cylindrical mask (gray) positioned above a four-helix bundle from the reconstruction of VelcroVax in the *T *= 4 arrangement. (B) All classes from focused classification, with the proportion of subparticles assigned to each class indicated. Classes are shown oriented in the same way as the mask shown in the inset in panel A. Download FIG S4, TIF file, 1.4 MB. Copyright © 2023 Kingston et al. 2023 Kingston et al. https://creativecommons.org/licenses/by/4.0/ This content is distributed under the terms of the Creative Commons Attribution 4.0 International license.

### Generation and capture of JUNV gp1.

**FIG 4:**
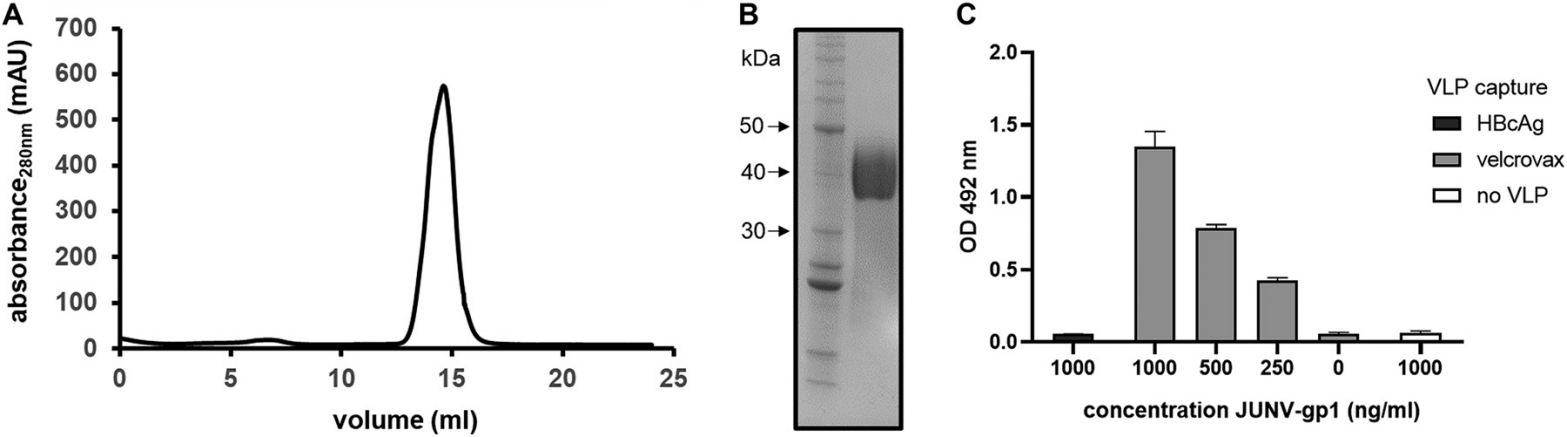
Generation of JUNV gp1 and interaction with VelcroVax. SUMO-tagged JUNV gp1 was produced in HEK293T cells and partially purified before processing through a final round of SEC. (A) Representative SEC elution profile for recombinantly derived JUNV gp1. (B) Reducing Coomassie-stained SDS-PAGE of SEC-purified JUNV gp1 with pertinent molecular mass standard sizes indicated in kDa. (C) ELISA was used to assess binding of HBcAg or VelcroVax to SUMO-tagged JUNV gp1. Particles coated on plates were subsequently incubated with JUNV gp1 and probed with anti-JUNV gp1 clone OD01-AA09, followed by incubation with anti-mouse HRP. Plates were incubated with OPD, and the OD was read at 492 nm and graphed as mean ± SEM; *n* = 3 in duplicate.

To determine whether VelcroVax retained a functional Affimer and thus was a suitable candidate for future immunization work, we investigated the ability of VelcroVax particles to capture a SUMO-tagged antigen. Given its importance as a target for neutralizing antibody responses (31–35), we elected to use the gp1 subcomponent of the arenavirus gp1 spike from JUNV as a candidate immunogen. We first produced and purified C-terminal SUMO-tagged JUNV gp1 from HEK293T cells. The glycoprotein was purified with successive rounds of IMAC and SEC (Fig. 4A), and SDS-PAGE followed by Coomassie blue staining verified the presence of glycoprotein within the peak fraction (Fig. 4B). The binding of SUMO-tagged JUNV gp1 to VelcroVax was assessed by indirect ELISA. After coating EIA plates with PBS, wt HBcAg VLPs or VelcroVax overnight, wells were blocked, and glycoprotein was added. A JUNV gp1-specific antibody was used to detect the glycoprotein within each well. No JUNV gp1 was detected in the wells coated with PBS or wt HBcAg. However, wells coated with VelcroVax bound JUNV gp1 in a concentration-dependent manner (Fig. 4C).

### Comparative immunization.

To compare the immunological consequences of immunization with free glycoprotein with that presented on VLPs, immunization trials were carried out in BALB/c mice. To this end, two groups of seven mice were immunized three times at 2-week intervals with JUNV gp1 mixed with wt HBcAg VLPs (to control for nonspecific effects that may be induced by the VLPs alone) or bound to VelcroVax. Immunizations were administered subcutaneously in the presence of 2.5 nmol CpG ODN1668, and serum samples were collected between boosts and 2 weeks after the final dose was administered (Fig. S5).

10.1128/msphere.00568-22.5 FIG S5 Immunization schedule. Two groups of seven female BALB/c mice were immunized three times at 2-week intervals with a total of 2 μg protein, according to the above schedule. Vaccines were composed of 1 μg JUNV gp1, 1 μg VLP (HBcAg or VelcroVax), and 2.5 nmol CpG ODN 1668 in a total volume of 200 μL. Intermittent blood samples were collected on days 13 and 27. At the conclusion of the experiment, mice were humanely euthanized, and blood was collected via cardiac puncture while animals were under terminal anesthesia. Download FIG S5, TIF file, 0.06 MB. Copyright © 2023 Kingston et al. 2023 Kingston et al. https://creativecommons.org/licenses/by/4.0/ This content is distributed under the terms of the Creative Commons Attribution 4.0 International license.

**FIG 5:**
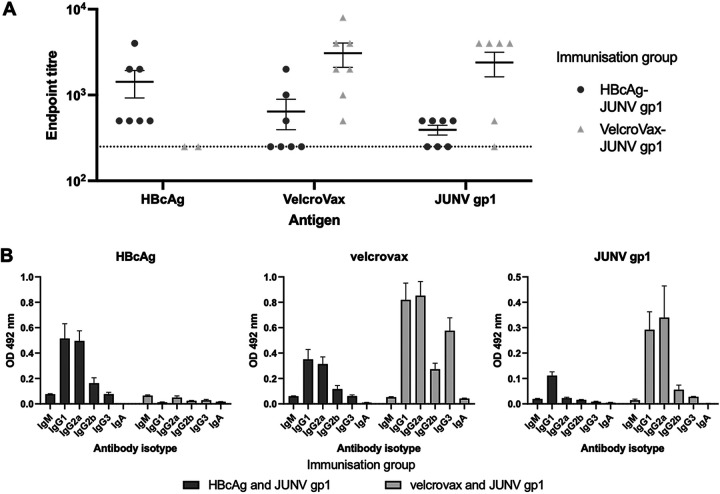
Reactive antibody titers and isotypes. (A) Antisera generated by immunization of mice with HBcAg and JUNV gp1 or VelcroVax and JUNV gp1 were assessed for endpoint titers with HBcAg, VelcroVax, and JUNV gp1. Sera were assessed at dilutions between 1:250 and 1:8,000 (HBcAg and VelcroVax) (*n* = 7 in duplicate) or 1:250 to 1:4,000 (JUNV gp1) (*n* = 7 twice in duplicate) and graphed as mean and SEM. Dotted line indicates the limit of detection (1:250). Titers were considered positive if the mean OD_492_ was ≥0.2 (approximately 2× the OD of preimmune serum). (B) Sera were subsequently assessed for isotype-specific reactivity with HBcAg, VelcroVax, and JUNV gp1. Sera were assessed at 1:125 dilution and graphed as mean and SEM; *n* = 7 in duplicate.

Serum samples collected at the completion of the immunization series were assessed for the presence of IgG antibodies directed against HBcAg, VelcroVax, and JUNV gp1 (Fig. 5A). All mice immunized with the wt HBcAg and JUNV gp1 generated antibodies reactive with HBcAg. Although VelcroVax VLPs retain one unmodified HBcAg monomer per subunit, the antibodies generated against wt HBcAg recognized VelcroVax particles less efficiently (*P* = 0.1883). Similarly, the group immunized with wt HBcAg and JUNV gp1 did not generate high-titer anti-gp1 antibodies compared to the VelcroVax gp1 immunization group (*P* = 0.0202). However, mice immunized with VelcroVax and JUNV gp1 generated antibodies that efficiently recognized JUNV gp1 and VelcroVax but not wt HBcAg (Fig. 5A).

To better understand the T helper (Th) bias of the immune responses generated, we carried out antigen-specific isotyping of immune sera. Plates were coated with antigen and blocked as described above. Antisera were added to wells and incubated before the addition of isotype-specific detection antibodies. Both HBcAg and VelcroVax induced high levels of both IgG1 and IgG2a, suggesting a balance between Th1 and Th2 type responses (Fig. 5B). Interestingly, despite the balanced response generated against HBcAg in the HBcAg immunization group, the unbound JUNV gp1-specific antibodies induced were almost exclusively IgG1, indicating a strong Th2 bias directed against the glycoprotein. In contrast, the anti-JUNV gp1 antibodies generated by VelcroVax-JUNV gp1 immunization were balanced between IgG1 and IgG2a (Fig. 5B), a potentially important characteristic for the development of effective viral vaccines.

No direct neutralization of pseudovirus was detected using sera from either immunization group at 1:100 dilution (Fig. S7A). At a higher serum concentration (1:10), limited neutralization was detected in some serum samples, and the VelcroVax group showed higher direct neutralization at 1:10 dilution compared to the wt HBcAg immunization group (*P* = 0.01), although mean neutralization was just 24.89%. Additionally, neutralization did not correlate with total reactive antibody titer (Fig. S7B and S7C) or isotype (qualitative).

10.1128/msphere.00568-22.7 FIG S7 Pseudovirus neutralization. JUNV pseudovirus was produced with a firefly luciferase reporter and used to transduce RD cells. (A) The ability of immune serum to directly neutralize 1 × 10^5^ RLU pseudovirus was assessed at 1:100 dilution. Data are graphed showing average RLU of duplicate wells from individual animals; mean RLU/50 μL (*n* = 7) ± SEM. (B and C) Sera were tested for pseudovirus neutralization at 1:10 dilution and graphed as percent neutralization relative to a nonserum containing control. Neutralization from individual animals was graphed against total JUNV gp1 reactive titer at 1:250 dilution (complete reactive titers in Fig. 5). Graphed mean values from duplicate pseudovirus neutralization wells from individual animals and mean OD_492_ from *n* = 3 duplicate JUNV gp1 ELISA are shown. Download FIG S7, TIF file, 0.6 MB. Copyright © 2023 Kingston et al. 2023 Kingston et al. https://creativecommons.org/licenses/by/4.0/ This content is distributed under the terms of the Creative Commons Attribution 4.0 International license.

## DISCUSSION

There is a global need for rapid development of vaccines that are adaptable to emerging pathogens and deliverable at low cost for use in LMICs. One approach to achieve this goal relies on the development of a common carrier protein modified to present different antigens. Thus, a single carrier may be used as the foundation for vaccines against a range of pathogens, reducing vaccine development time and cost. To this end, we synthesized a carrier nanoparticle based on the HBcAg protein, containing an adapter sequence to allow the postpurification coupling of antigens to VLPs. We propose that this nanoparticle can form the basis of a modifiable vaccine strategy.

The recombinant expression of HBcAg protein in a variety of prokaryotic and eukaryotic systems and its assembly into stable and highly immunogenic HBcAg VLPs were first demonstrated in the 1980s (28, 36). Subsequently, a number of studies have demonstrated its potential as a chimeric vaccine delivery system, but it has yet to be developed as a component of licensed vaccines. HBcAg VLPs are formed from monomers assembled into dimers, with 90 (*T *= 3) or 120 (*T *= 4) of these dimers assembling to form particles approximately 30 and 34 nm in diameter, respectively (37). These particles are arranged with external facing N termini, a long helical region followed by a flexible surface exposed loop (MIR), and another helical region leading to an internal facing C-terminal end (Fig. 1A). The genetic fusion of two monomers results in a tandem HBcAg construct (36) and the introduction of an anti-SUMO Affimer (27) into the first MIR of this tandem construct forms the basis of our VLP capture system, VelcroVax (Fig. 1B). Expression of similarly modified constructs has previously been described in plants and bacterial expression systems; here, we have used P. pastoris (28). Expression of HBcAg or VelcroVax in P. pastoris results in the efficient formation of VLPs, yielding approximately 300 μg of purified VelcroVax per 100 mL of yeast culture. Additionally, both HBcAg and VelcroVax particles have diameters consistent with the formation of both *T *= 3 and *T *= 4 symmetric particles (Fig. 1C; Fig. S1).

There are several published structures of wt and mutant HBcAg particles; however, no high-resolution structures exist of tandem HBcAg VLPs. Using VelcroVax particles produced in P. pastoris, we generated high-resolution structures of *T *= 3* and *T *= 4 symmetric particles, with the proportions of both particle configurations found to be approximately equal. The *T *= 3* reconstruction was less well resolved than the *T *= 4 reconstruction, likely because 5-fold symmetry was imposed during refinement to account for the unique symmetrical arrangement of *T *= 3* particles (Fig. 2; Fig. S2 and S3A). Both the *T = *3* and *T *= 4 reconstructions had clearly resolved density for residues corresponding to the helices of both HBcAg molecules within the tandem VelcroVax sequence. Unsurprisingly, given the presence of flexible linking sequences, the SUMO-Affimer, the second MIR, and the internal Gly-Ser-linker lacked clearly defined density. The fact that each fused dimer could occupy one of two orientations, leading to four unique arrangements per asymmetric unit for the *T *= 4 particle, also likely contributed to the poorly defined density of these regions (Fig. S3B). Focused classification yielded no improvement in Affimer density, and particles were distributed relatively evenly between focused classes, suggesting a high level of variability and flexibility in this region, as expected (Fig. S4). When a low-pass filter was applied to both *T *= 3* and *T *= 4 reconstructions, amorphous density was present above the four-helix bundles of the capsid, consistent with the presence of the Affimer (Fig. 3). Given the difficulty in resolving flexible/mobile regions of the VLP at high resolution, we were unable to determine structurally whether Affimers displayed on the surface of particles retained a native fold.

To determine whether Affimers expressed in the context of VLPs retained functionality, we mixed SUMO-tagged JUNV gp1 with VelcroVax and assessed binding by ELISA (Fig. 4). After confirming binding between VelcroVax VLPs and JUNV gp1, we carried out an immunization trial using the complexed particles. The gp1 of JUNV forms a subcomponent of the trimeric gp spike displayed on the envelope surface and facilitates recognition of transferrin receptor 1 during host-cell entry (38, 39). Although virus-neutralizing antibodies reactive with gp1 have been described (indeed the monoclonal antibody used in this study to detect the protein is reported to neutralize the virus), only low levels of neutralizing activity have been observed even after multiple immunizations in the presence of strong adjuvants such as Freund’s complete adjuvant (35, 40). Despite the apparent poor immunogenicity of the protein, we investigated whether the presence of JUNV gp1 in the context of a nanoparticle vaccine may improve the maturation potential of antibodies directed against the glycoprotein subunit.

The size of nanoparticles (30 to 100 nm) has previously been shown to improve DC-mediated recognition, uptake, and enhanced antigen presentation (16, 23, 24), and the repetitive structure of these nanoparticles can enhance the cross-linking of receptors on B cells, which functionally improves signaling and is coupled with a shift in the cytokine milieu leading to a more balanced Th1/Th2 type response (26). While the antibodies generated here were poorly neutralizing in a pseudovirus system (Fig. S7), the coupling of JUNV gp1 to VelcroVax both increased anti-JUNV gp1 antibody titers and generated a balanced Th1/Th2 response (Fig. 5). As is the case with most peptide immunogens, this balance was not observed for the uncoupled JUNV gp1 immunization group, where anti-gp1 antibodies were predominantly IgG1 (Th2), despite the anti-HBcAg response from the same group showing a Th1/Th2 balance (Fig. 5B). Together these data suggest that the VelcroVax platform generated here is both suitable at improving total reactive titers as well as balancing the Th bias, a property that is likely to be useful for future glycoprotein vaccine candidates.

Importantly, the broad response generated by the VelcroVax-JUNV gp1 complex indicates a more effective presentation of the target antigen compared to VLP with unbound antigen. This broad response is generally desirable and may contribute to immunological protection in the absence of efficient direct neutralization (41, 42). Further immunological studies would be needed to fully evaluate the VelcroVax platform. We therefore propose that the VelcroVax platform offers an adaptable system for future VLP vaccines and may be a useful tool for the generation of rapid-response vaccines in the future.

## MATERIALS AND METHODS

### Generation of HBcAg VLPs in yeast.

Genes encoding either HBcAg or VelcroVax were introduced downstream of the AOX1 promoter within the pPinkHC expression vector (ThermoFisher Scientific). The VelcroVax sequence consists of a fused HBcAg dimer with the SUMO-Affimer sequence introduced within the first major immunodominant region (MIR) of this dimer. A Gly-Ser linking sequence was used to provide flexibility to this domain, and for consistency this linker was present in all HBcAg subunits used here. Similar to previously described protocols (43), plasmids were linearized with *Afl*II and electroporated into PichiaPink strain 1 (Invitrogen), and then transformed yeast were plated on adenine dropout media and incubated at 28°C for 3 to 5 days. To screen for expression, colonies were selected at random and inoculated into 5 mL YPD media (10 g/L yeast extract, 5 g/L peptone, 20 g/L dextrose) before incubation at 28°C, 250 rpm for 48 h. Cells were pelleted at 1,500 rcf and resuspended in 1 mL YPM (10 g/L yeast extract, 5 g/L peptone, 2% vol/vol methanol). Cultures were incubated at 28°C, 250 rpm for 72 h, and supplemented with 1 or 2% vol/vol methanol every 24 h (VelcroVax and HBcAg expression, respectively). Cells were collected at 48 h and assessed for protein production by Western blot.

For large-scale production, a glycerol stock of VelcroVax- or HBcAg-expressing P. pastoris was used to inoculate 5 mL YPD and incubated at 28°C for 48 h at 250 rpm before inoculation into 200 mL of YPD and incubation for a further 48 h at 28°C, 250 rpm. Cells were pelleted at 1,500 rcf and resuspended in 200 mL YPM (1 or 2% vol/vol methanol, as above) before incubation at 28°C, 250 rpm for 72 h. Media were supplemented with methanol every 24 h. Cells were pelleted at 4,000 rcf and resuspended in 30 mL EDTA-free breaking buffer (50 mM Na_3_PO_4_, 5% vol/vol glycerol, pH 7.4) with cOmplete EDTA-free protease inhibitor cocktail (Roche).

### VLP purification and quantitation.

To isolate VLPs from P. pastoris, cells were disrupted at 40 kpsi and supplemented with 1 mM MgCl and 250 units denarase (c-LEcta) before incubation at room temperature for 2 h with agitation. Samples were clarified at 4,000 rcf, and clarified supernatant was precipitated overnight at 4°C with 20% vol/vol saturated ammonium sulfate solution (structural studies) or 8% wt/vol PEG-8000 (immunogenicity and antigenicity studies). Precipitated material was pelleted at 4,000 rcf for 30 min and resuspended in 30 mL PBS. Insoluble material was removed by centrifugation at 10,000 rcf. The soluble material was pelleted through a 30% sucrose cushion at 150,000 rcf for 3.5 h. Pellets were resuspended in 1 mL PBS and separated on a 15 to 45% sucrose gradient at 50,000 rcf for 12 h. One-milliliter fractions were collected manually (top down) and assessed for the presence of HBcAg-reactive proteins by Western blot with MAb 10E11 using standard protocols. The protein content of fractions was assessed directly by BCA assay (Pierce, ThermoFisher Scientific), or the VLPs were concentrated and the buffer was exchanged using 100K MWCO PES concentrator columns (Pierce, Thermo Scientific) before quantification by BCA assay. To purify VLPs for structural analysis, this protocol was slightly modified, as described by Snowden et al. (44).

### Electron microscopy.

To prepare samples for negative stain EM, carbon-coated 300-mesh copper grids (Agar Scientific, UK) were glow-discharged under air (10 mA, 30 s) before application of a 3-μL sample for 30 s. Excess liquid was wicked away, and then grids were washed two to four times with 10 μL distilled H_2_O. Staining was then performed with 1 to 2% uranyl acetate solution (UA). UA was applied (10 μL) and immediately wicked away, and then an additional 10 μL UA was applied and allowed to incubate for 30 s before being blotted and left to air dry. Imaging was performed using either (i) an FEI Tecnai G2-spirit with LaB_6_ electron source, operating at 120 kV and equipped with a Gatan Ultra Scan 4000 CCD camera, with a calibrated object sampling of 0.48 nm/pixel; or (ii) an FEI Tecnai F20 with field emission gun, operating at 200 kV and equipped with an FEI CETA camera, with a calibrated object sampling of 0.418 nm/pixel.

For cryoEM, samples were vitrified as described by Snowden et al. (44). Briefly, ultrathin continuous carbon-coated lacey carbon 400-mesh copper grids (Agar Scientific, UK) were glow discharged in air (10 mA, 30 s), and then 3-μL samples were applied to the grid surface for 30 s in a humidity-controlled chamber (8°C, 80% relative humidity). Excess liquid was removed by blotting (1.0 to 4.0 s) before plunge freezing in liquid nitrogen-cooled liquid ethane using a LEICA EM GP plunge freezing device (Leica Microsystems, Germany). Imaging was performed using an FEI Titan Krios transmission EM (ABSL, University of Leeds) operating at 300 kV, with a calibrated object sampling of 1.065 Å/pixel. Full data collection parameters are provided in Table S1.

10.1128/msphere.00568-22.9 TABLE S1 CryoEM data collection parameters for VelcroVax. Download Table S1, TIF file, 1.2 MB. Copyright © 2023 Kingston et al. 2023 Kingston et al. https://creativecommons.org/licenses/by/4.0/ This content is distributed under the terms of the Creative Commons Attribution 4.0 International license.

### Image processing.

Image processing was performed using the Relion 3.0 and Relion 3.1 pipelines (45, 46). MotionCor2 (47) was used to correct any motion-induced blurring in raw micrographs, and then CTF parameters were estimated using Gctf (48). A small subset of VLPs (both *T *= 4 and *T *= 3*) was manually selected and used to generate two-dimensional (2D) class averages, used as templates for automated picking of the entire data set. Initially, ~250,000 particles (including contaminants and erroneously selected areas of carbon) were extracted and 2× down-sampled for 2D classification, with CTFs ignored until the first peak. All classes resembling VLPs (~130,000 particles) were taken forward for additional 2D classification without CTFs ignored until the first peak, at which point two independent particle stacks were created and reextracted without down-sampling: one for *T *= 4 VLPs and one for *T *= 3* VLPs (each containing ~50,000 particles). Three-dimensional (3D) refinement was performed separately for each particle stack, based on initial models generated *de novo* in Relion, with symmetry imposed (I1 for *T *= 4, C5 for *T *= 3*). Where appropriate, map resolution and quality were improved by iterative cycles of CTF refinement, Bayesian polishing, and 3D refinement with a solvent mask applied and flattened Fourier shell correlation (FSC) calculations. Maps were sharpened using a solvent-excluding mask, a nominal resolution was determined using the “gold standard” FSC criterion (FSC = 0.143) (Fig. S2; Table S2), and then local resolution was calculated and a local resolution-filtered map generated in Relion.

10.1128/msphere.00568-22.10 TABLE S2 Quantitative parameters and validation statistics related to cryoEM image processing and model building. Download Table S2, TIF file, 0.1 MB. Copyright © 2023 Kingston et al. 2023 Kingston et al. https://creativecommons.org/licenses/by/4.0/ This content is distributed under the terms of the Creative Commons Attribution 4.0 International license.

For *T *= 4 VLPs, focused classification was performed in an attempt to resolve Affimer density, using a protocol described previously (44, 49–51). Briefly, SPIDER (52) was used to generate a cylindrical mask that was manually placed above a four-helix bundle using the University of California—San Francisco (UCSF) Chimera (53). A soft edge was added to the mask in Relion. *T *= 4 VLP particles and their associated orientational information from a symmetrized 3D refinement were used to generate a symmetry-expanded particle stack using the relion_particle_symmetry_expand tool. These data were then subjected to masked 3D classification without alignments, with a regularization parameter of 40.

### Model building and refinement.

Atomic models were built into the density maps for both *T *= 4 and *T *= 3* VLPs. First, a homology model was generated using SWISS-MODEL (54). Copies of this model were fitted into density for each quasiequivalent position within the *T *= 4 and *T *= 3* VLP asymmetric units using UCSF Chimera (53), and unresolved segments of the peptide backbone were removed. Models were then inspected and manually refined in Coot (55) before automated refinement in Phenix (56) to improve model-to-map fit and atomic geometry. This process was repeated iteratively, with at least one iteration performed with a symmetrized atomic model to avoid erroneous placement of atomic coordinates in density from adjacent asymmetric units. Model validation (Table S2) was performed using MolProbity (57).

### Structure analysis and visualization.

Visualization of structural data was performed in UCSF Chimera (53), UCSF ChimeraX (58), and PyMOL (The PyMOL Molecular Graphics System, Version 2.1, Schrödinger, LLC). RMSD calculations were performed using the “MatchMaker” tool in UCSF Chimera, with default settings.

### Generation of recombinant JUNV gp1.

The sequence encoding amino acids 87 to 231 of JUNV gp1 (GenBank ACO52428) was PCR-amplified and cloned into a pHLsec vector (59) containing a C-terminal SUMO tag (GenBank AVL26008.1) and hexahistidine tag. The JUNV gp1-SUMO construct was transfected into human embryonic kidney (HEK) 293T cells, grown in roller bottles for transient expression (60). Four days posttransfection, cell supernatant was supplemented with NaCl (700 mM), Tris pH 8.0 (20 mM), and imidazole (15 mM). JUNV gp1 was purified by immobilized metal affinity chromatography, using a 5-mL HisTrap Excel column (Cytiva), followed by size exclusion chromatography (SEC) with a Superdex 200 increase 10/300 GL column (Cytiva) equilibrated with 15 mM Tris (pH 8.0), 200 mM NaCl, and 0.5 mM EDTA. The JUNV gp1 containing peak was further purified over a 1-mL HiTRAP Q (HP) column (Cytiva) using a 30 mM Tris pH 8.0 running buffer and a linear,0 to 500 mM NaCl gradient. The JUNV gp1 was repurified by SEC (as above). Following concentration, protein samples were snap frozen and stored at −80°C.

### ELISA to detect antigen capture.

The capture of SUMO-tagged JUNV gp1 by VelcroVax was assessed by ELISA. Plates were coated with 50 μL 2 μg/mL of wt HBcAg VLP, VelcroVax, or PBS and incubated overnight at 4°C. Plates were blocked with 2% skim-milk powder in PBS 0.1% Tween 20; JUNV gp1 was added to wells at 1,000, 500, and 250 ng/mL; and PBS was used as a negative control. Plates were incubated at 37°C for 1 h before being washed. The presence of JUNV gp1 was determined using a 1:2,000 dilution of mouse virus-neutralizing anti-JUNV gp1 (obtained through BEI Resources, NIAID, NIH: monoclonal anti-Junín virus, clone OD01-AA09 [immunoglobulin G, mouse], NR-2567). After incubation, plates were washed and 50 μL of anti-mouse horseradish peroxidase (HRP) was added to wells (Sigma). Plates were incubated for a further hour at 37°C, before a final wash step and the addition of 100 μL/well Sigmafast OPD (Sigma). After 15 min, 50 μL of 3 M HCl was added to wells to stop the reaction and the optical density was determined at 492 nm (OD_492_). Data were graphed as mean OD_492_ with SEM (GraphPad Prism).

### Immunization.

Groups of seven female BALB/c mice were purchased from Charles River UK at 5 weeks of age. Mice were housed for 2 weeks before the initiation of experimental procedures, at which point samples of preimmune sera were collected (approximately 50 μL total blood volume) via the tail vein. Mice were then immunized three times at 2-week intervals subcutaneously in the rear upper flank with a total volume of 100 μL per dose. Vaccines were composed of 1 μg of VLP (HBcAg or VelcroVax) (Fig. S6) and 1 μg of JUNV gp1 in the presence of 2.5 nmol of the murine TLR9-stimulatory molecule CpG ODN1668 (Invivogen). Samples were assembled 24 h preimmunization to facilitate SUMO-linked conjugation of JUNV gp1 to VLP and stored at 4°C until used. All vaccine components were tested for endotoxin content and immunizations contained less than 2.5 EU/dose (Pierce LAL Chromogenic Endotoxin Quantitation kit; Thermo Scientific). Serum samples were collected on days 13 and 27 (as above) (Fig. S5). On day 41, final blood samples were taken via cardiac puncture while mice were euthanized under sodium pentobarbitone. All animals were housed under specific pathogen-free conditions and monitored for well-being. All animal procedures were performed in strict accordance with UK Home Office guidelines, under license PP2876504 granted by the Secretary of State for the Home Office, which approved the work described, in accordance with local ethical guidelines and internal committee approval for animal welfare at NIBSC. This study conforms to all relevant ethical regulations for animal work in the United Kingdom. We elected to use seven animals in this trial due to the sensitivity of our assays to detect minor differences in immune outcomes between animals, and the moderate variability of responses generated within immunization groups, offering good statistical power and minimizing animal use.

10.1128/msphere.00568-22.6 FIG S6 Vaccine purity. HBcAg and VelcroVax vaccine samples were assessed by Coomassie blue stain (left) and anti-HBcAg-specific Western blot using monoclonal antibody 10E11. Download FIG S6, TIF file, 0.1 MB. Copyright © 2023 Kingston et al. 2023 Kingston et al. https://creativecommons.org/licenses/by/4.0/ This content is distributed under the terms of the Creative Commons Attribution 4.0 International license.

### Antibody titration and isotyping.

Antibody titers were assessed by indirect ELISA. To this end, 96-well EIA plates were coated with 50 μL 2 μg/mL target protein. Serum samples were assessed against HBcAg, VelcroVax, and JUNV gp1. Plates were blocked with 2% skim-milk powder in PBS 0.1% Tween 20 before the addition of duplicate dilutions of antisera at 1:250 to 1:4,000 or a PBS-only negative control and incubated at 37°C for 1 h. Plates were washed and 50 μL of rabbit anti-mouse HRP was added at 1:2,000 dilution (Sigma). Plates were incubated at 37°C for 1 h, washed, and incubated with 100 μL Sigmafast OPD (Sigma) for 15 min. Reactions were stopped with the addition of 50 μL 3 M HCl at OD_492_. Titers were considered positive when the mean OD_492_ was ≥0.2, which is ~2× the OD_492_ of preimmune serum in these assays. Data were graphed as mean and SEM and analyzed using an unpaired *t* test (GraphPad Prism).

To determine the isotypes of antibodies generated by immunization, plates were coated and blocked, as above. Sera were diluted 1:125, and 50 μL of sera or a PBS-only negative control was added to duplicate wells, before incubation at 37°C for 1 h. Plates were washed and 50 μL of isotype-specific goat anti-mouse antibody was added at 1:1,000 dilution (Sigma). Plates were incubated at 37°C for 1 h before being washed and addition of 50 μL of anti-goat HRP (Sigma). After a final 1-h incubation, plates were washed and developed with OPD, as above. The OD_492_ of negative-control wells (no sera) was deducted from the isotype-specific signal, and the mean OD was graphed on a bar chart (GraphPad Prism).

### Generating pseudovirus.

Previously described protocols were used to generate JUNV pseudovirus with slight modification (61). Briefly, HEK293T/17 cells were seeded at 30% density and incubated overnight to allow growth to 50 to 60% confluence at the time of transfection. The following day, DNA transfections were carried out by combining 1 μg p8.91 plasmid, with 1.5 μg Pcsflw (62) and 1.5 μg of pCAGGS-JUNV gp in 100 μL of Opti-MEM (Gibco) in a standard microcentrifuge tube, separately 12 μL of 1 mg/mL 25,000 MW linear PEI was diluted in 100 μL of Opti-MEM (Gibco). Tubes were incubated at room temperature for 5 min before PEI mix was added to DNA. The combined mixture was incubated at RT for 15 min before being added dropwise to culture media. Plates were incubated for 72 h at 37°C, 5% CO_2_, at which point the medium was harvested and filtered through a 0.45-μm PES filter.

### Pseudovirus titration.

Harvested cell supernatant containing JUNV pseudovirus was titrated as previously described (with slight modification) using RD cells (61). Briefly, in a 96-well white plate (Greiner Bio-One), 50 μL of pseudovirus-containing supernatant was added per well following a 2-fold serial dilution. Dilutions were added to wells containing 1 × 10^4^ RD cells/well and incubated for 72 h at 37°C, 5% CO_2_. The relative luminescence units (RLUs) were measured using the Bright-Glo (Promega) luciferase system.

### Neutralization assay.

Triplicate wells of diluted serum and 1 × 10^5^ RLU (equivalent after transduction) of JUNV pseudovirus were added to wells of a 96-well white opaque plate in a final volume of 100 μL. Plates were incubated for 1 h at 37°C, 5% CO_2_ in a humidified incubator, and 1 × 10^4^ RD cells were added to each well. Plates were incubated for 72 h before RLU was recorded, as above. For 1:100 diluted serum, raw data are graphed, and for 1:10 diluted samples, percent neutralization was determined relative to positive (no antibody, 0% neutralization) and negative (no pseudovirus, 100% neutralization) wells. The assay was verified using anti-JUNV gp1 clone OD01-AA09 (Fig. S8).

10.1128/msphere.00568-22.8 FIG S8 Pseudovirus neutralization with monoclonal anti-Junín virus antibody OD01-AA09. JUNV pseudovirus was produced with a firefly luciferase reporter and lentiviral gag-pol, and transduction was carried out in RD cells. Raw data of JUNV pseudoviral neutralization by monoclonal anti-Junín virus antibody OD01-AA09 are shown. Download FIG S8, TIF file, 0.1 MB. Copyright © 2023 Kingston et al. 2023 Kingston et al. https://creativecommons.org/licenses/by/4.0/ This content is distributed under the terms of the Creative Commons Attribution 4.0 International license.

### Data availability.

Atomic coordinates and density were uploaded to the Protein Data Bank (PDB) and Electron Microscopy Data Bank (EMDB) with the following deposition IDs: *T* = 3* - PDB 7ZQA, EMD-14868; *T* = 4 - PDB 7ZQ8, EMD-14866.
