# Supplementary figures and images for: VelcroVax: a “Bolt-On” Vaccine Platform for Glycoprotein Display
Source: mSphere. 2023 Jan 31;8(1):e00568-22. doi: 10.1128/msphere.00568-22 (PMC9942589; doi:10.1128/msphere.00568-22)

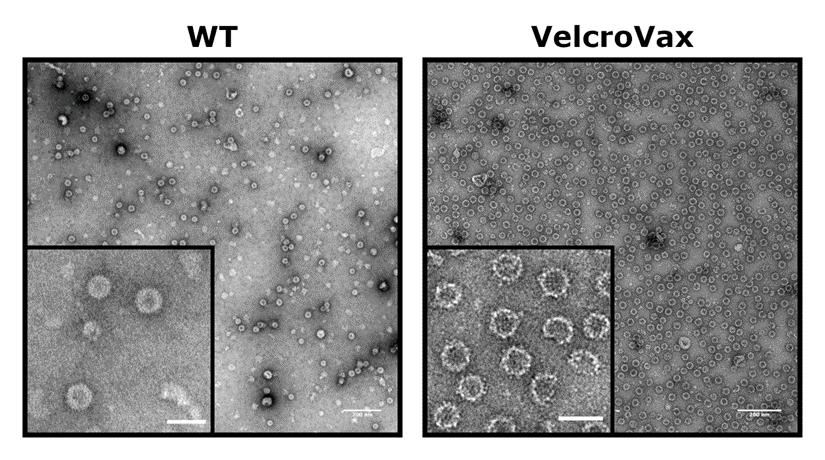

Supplement: FIG S1 [file msphere.00568-22-s0001.tif]

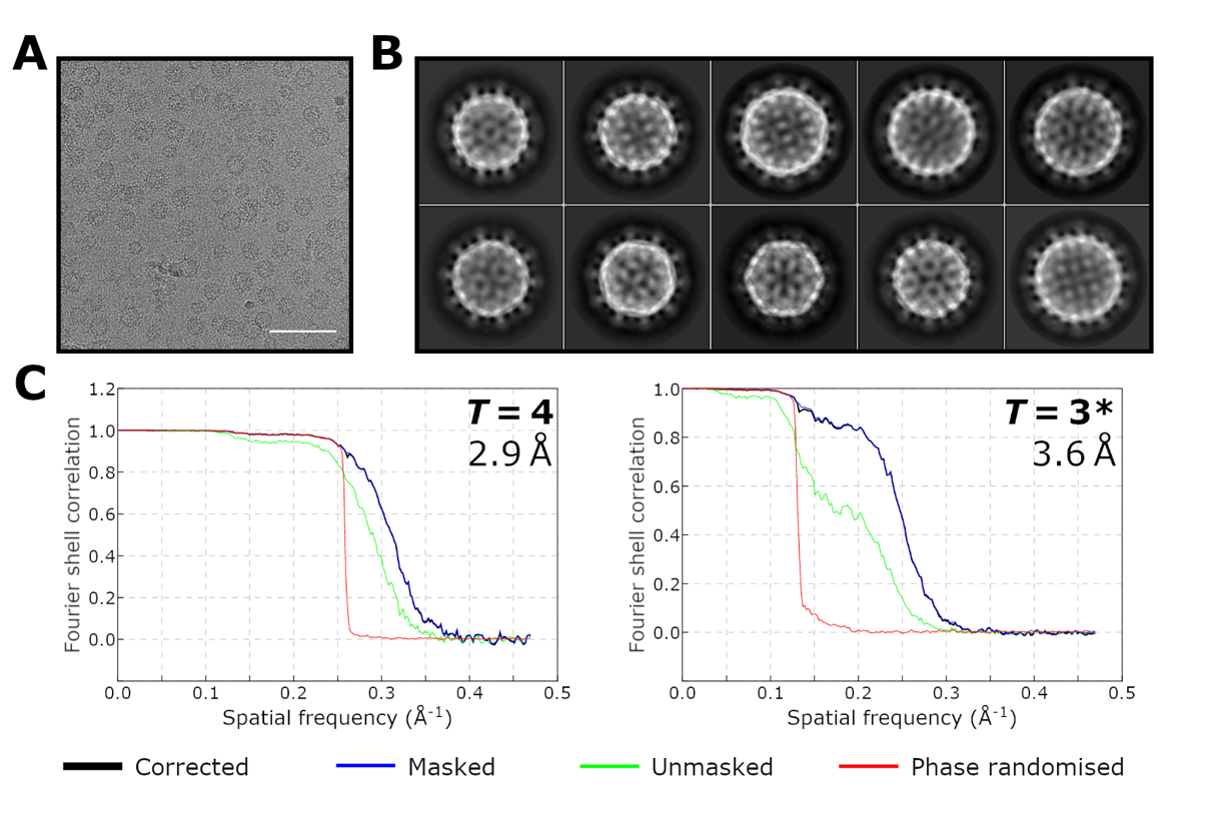

Supplement: FIG S2 [file msphere.00568-22-s0002.tif]

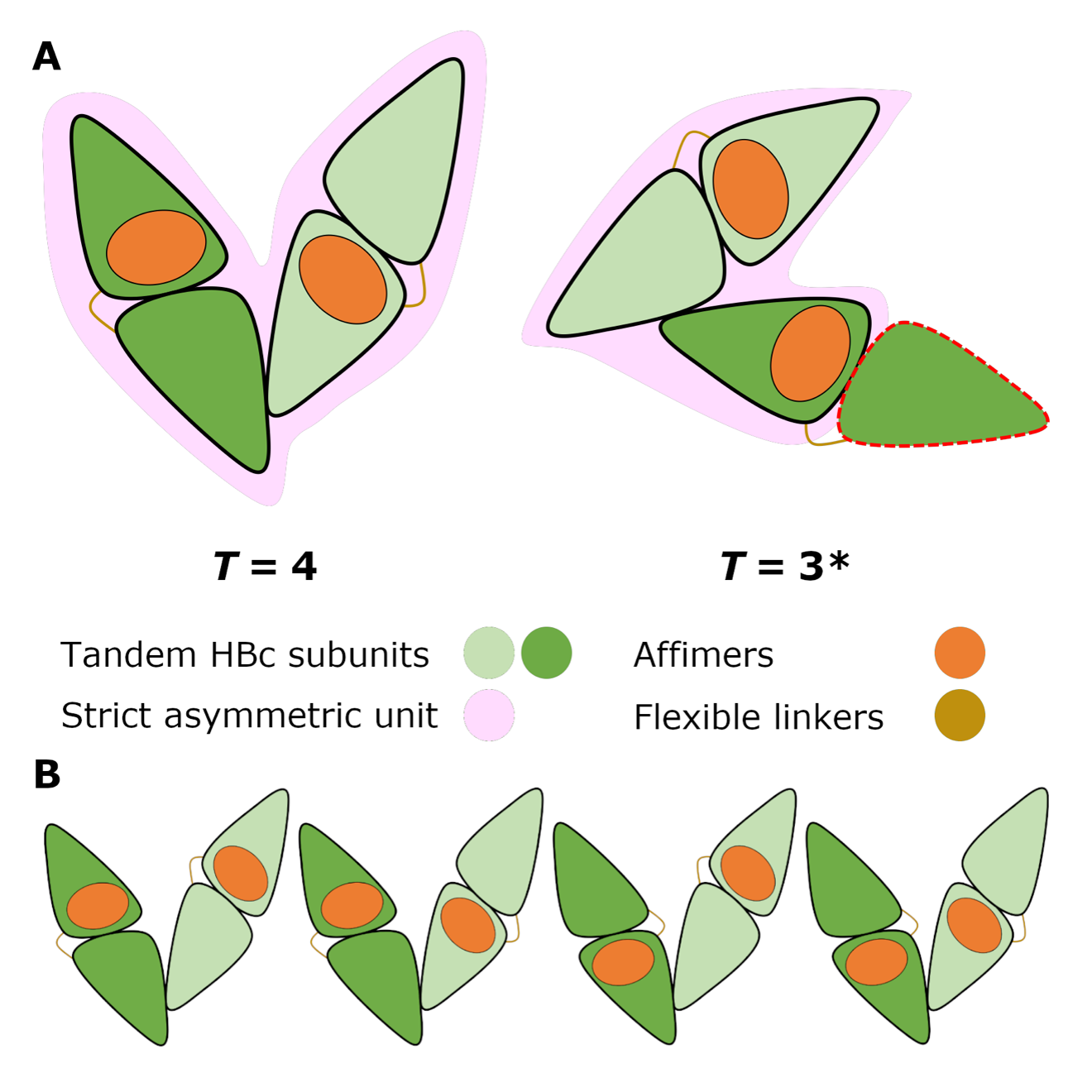

Supplement: FIG S3 [file msphere.00568-22-s0003.tif]

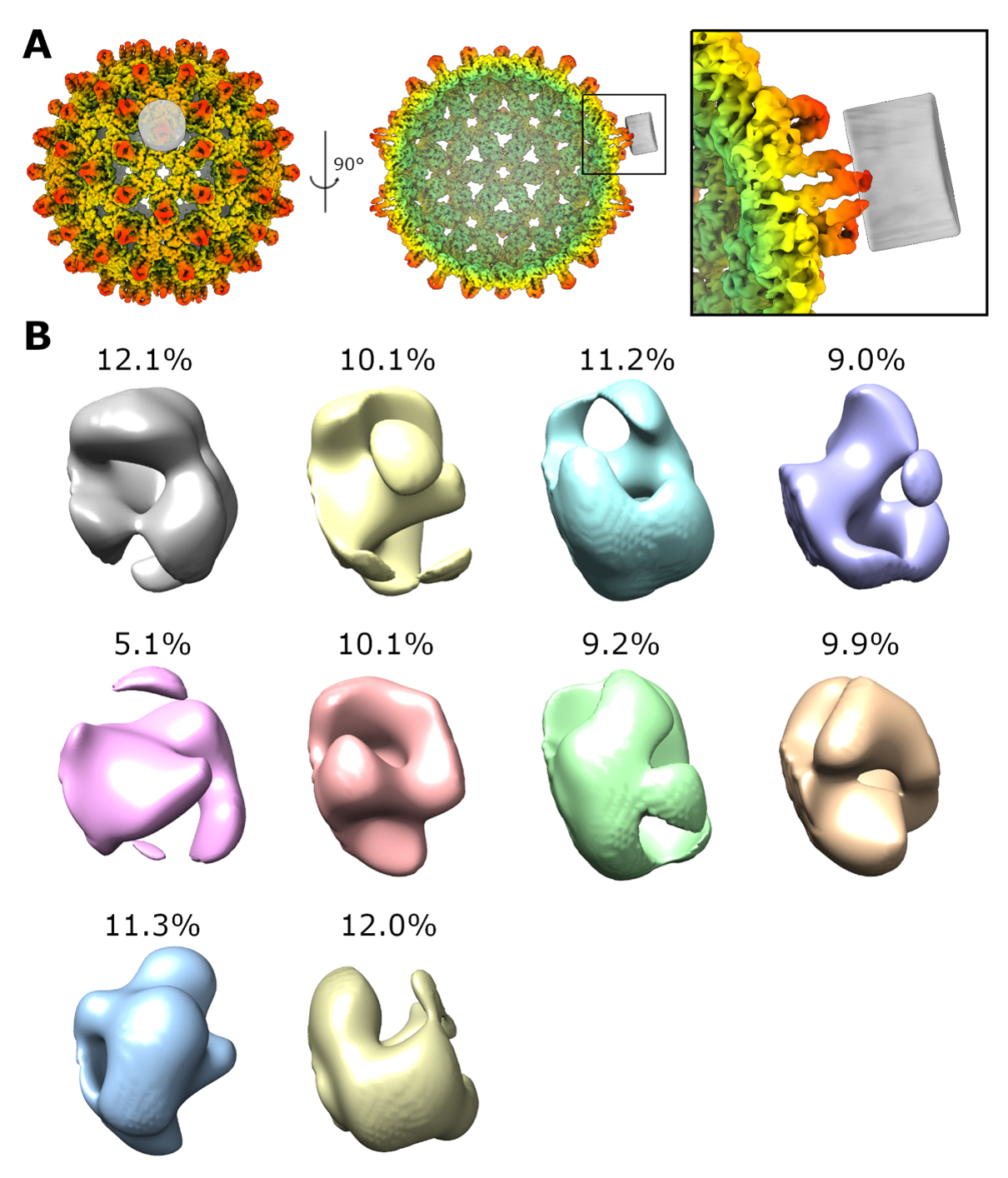

Supplement: FIG S4 [file msphere.00568-22-s0004.tif]

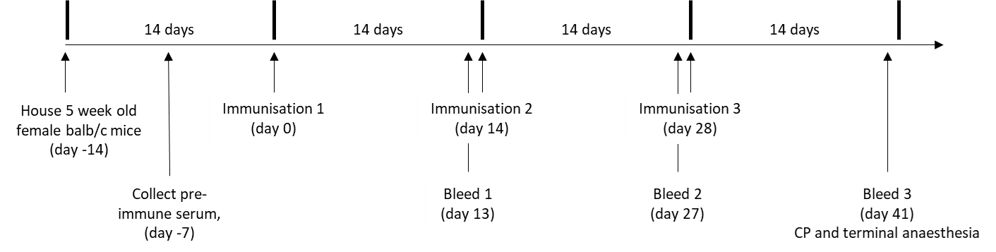

Supplement: FIG S5 [file msphere.00568-22-s0005.tif]

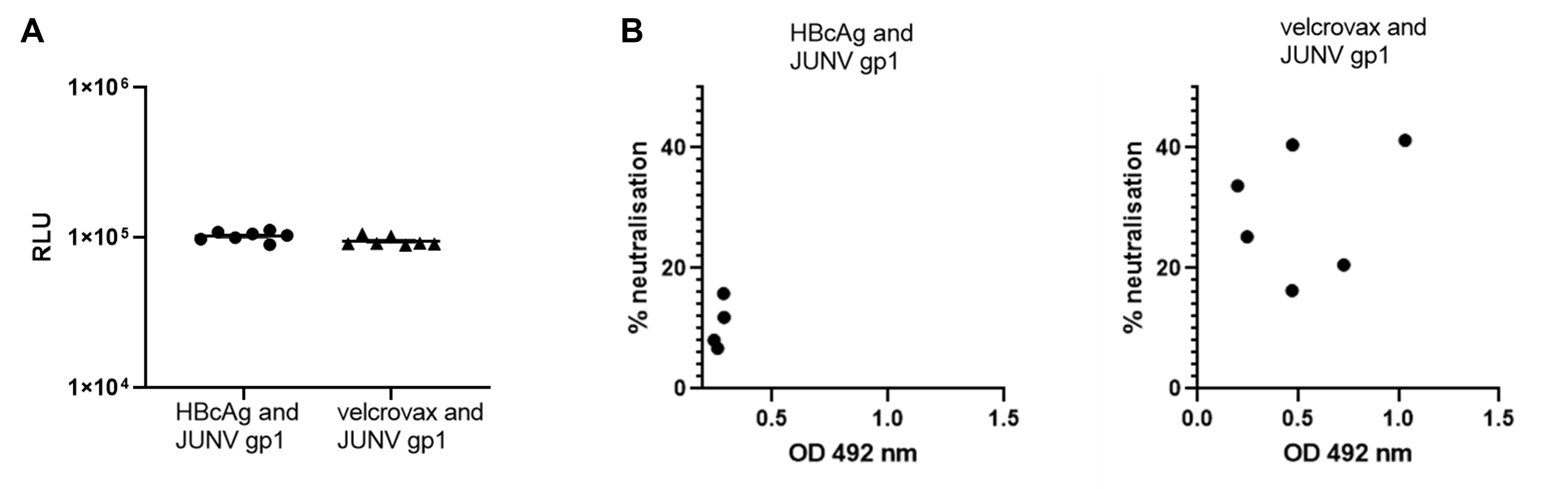

Supplement: FIG S7 [file msphere.00568-22-s0007.tif]

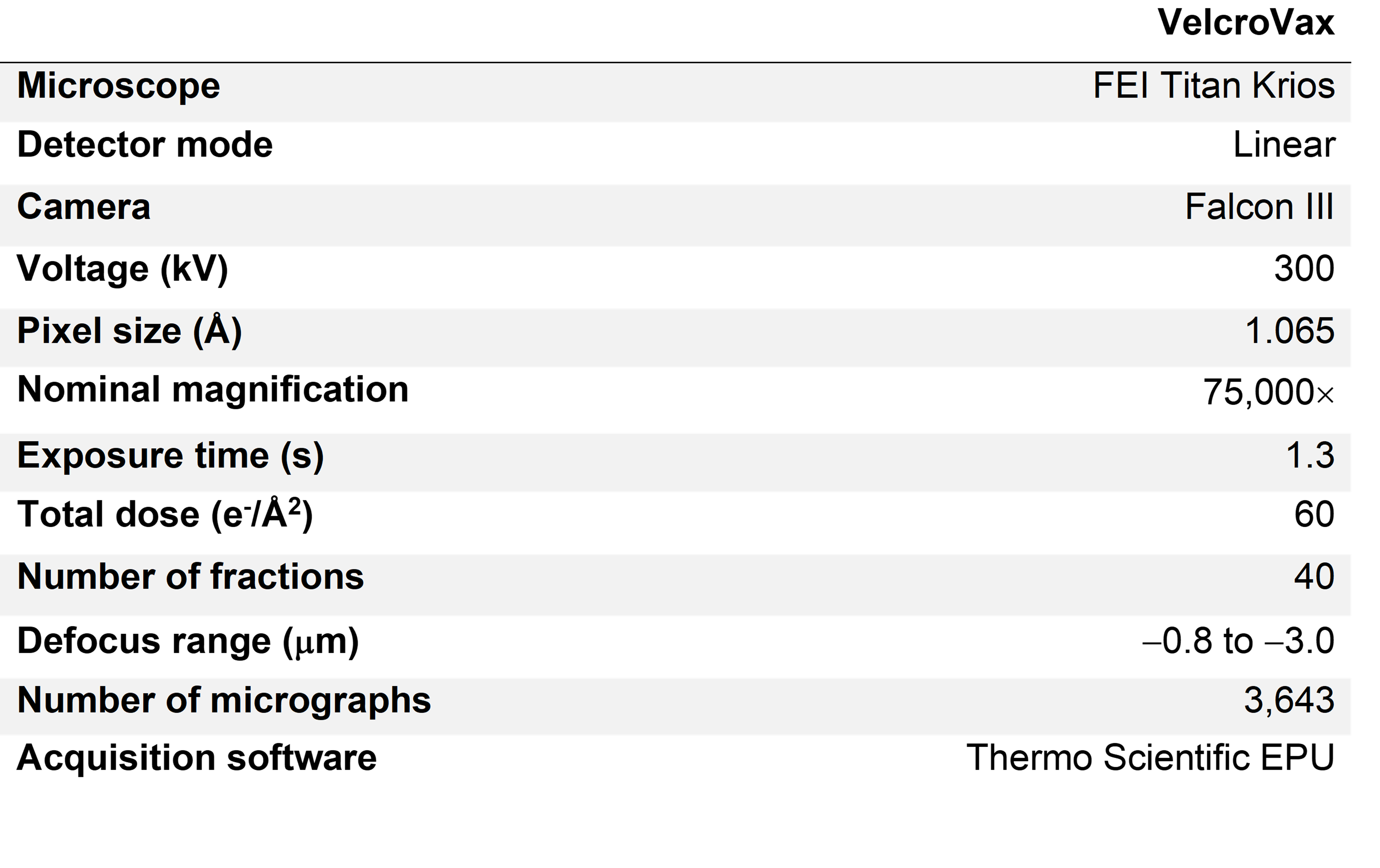

Supplement: TABLE S1 [file msphere.00568-22-s0009.tif]

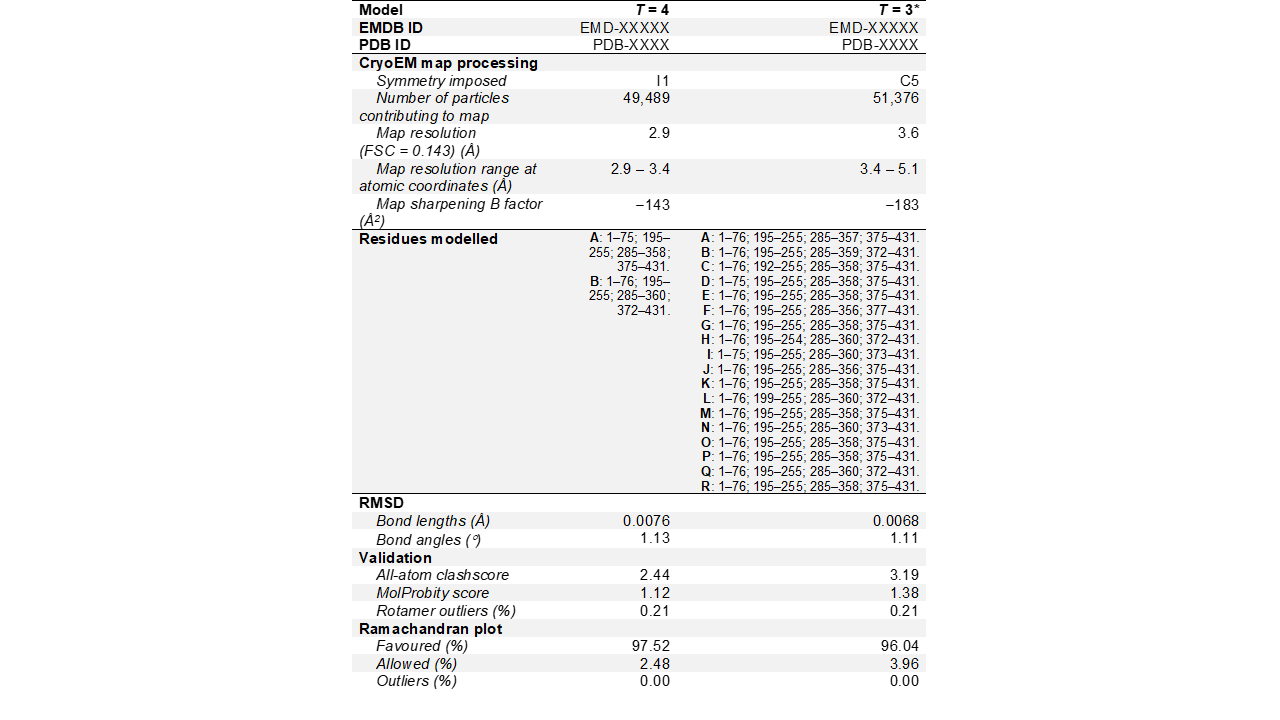

Supplement: TABLE S2 [file msphere.00568-22-s0010.tif]

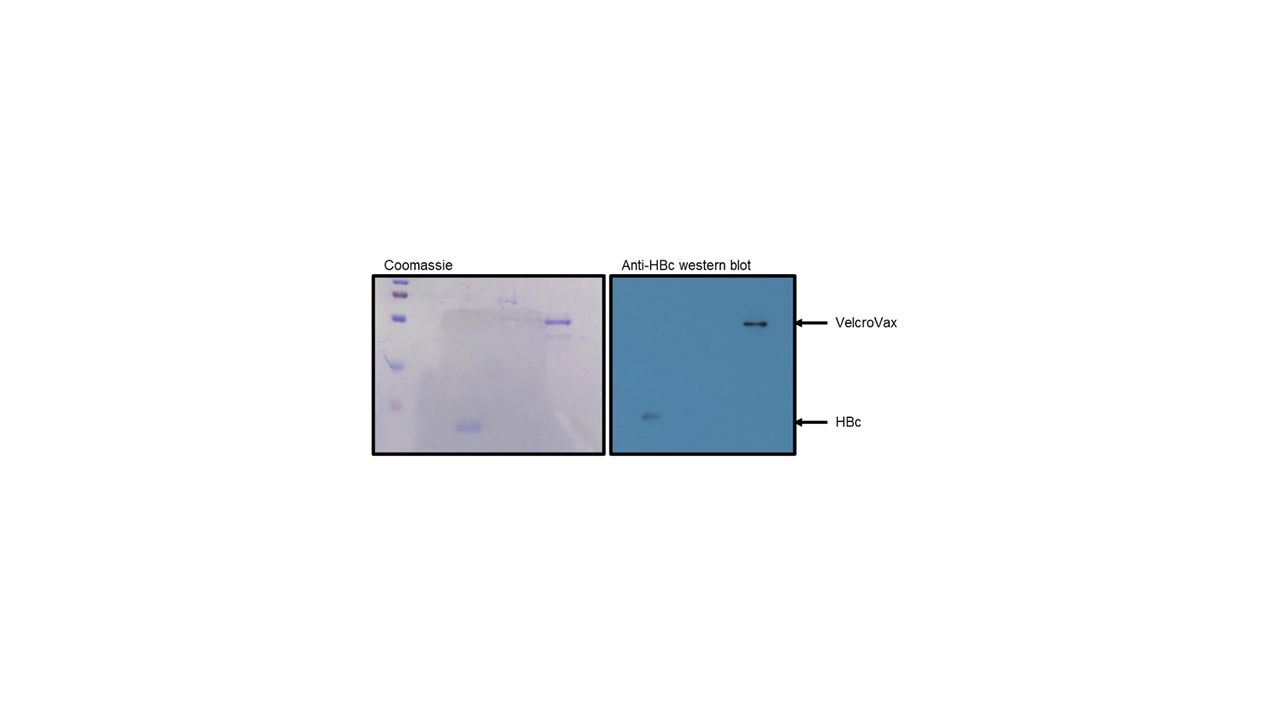

Supplement: FIG S6 [file msphere.00568-22-s0006.tif]

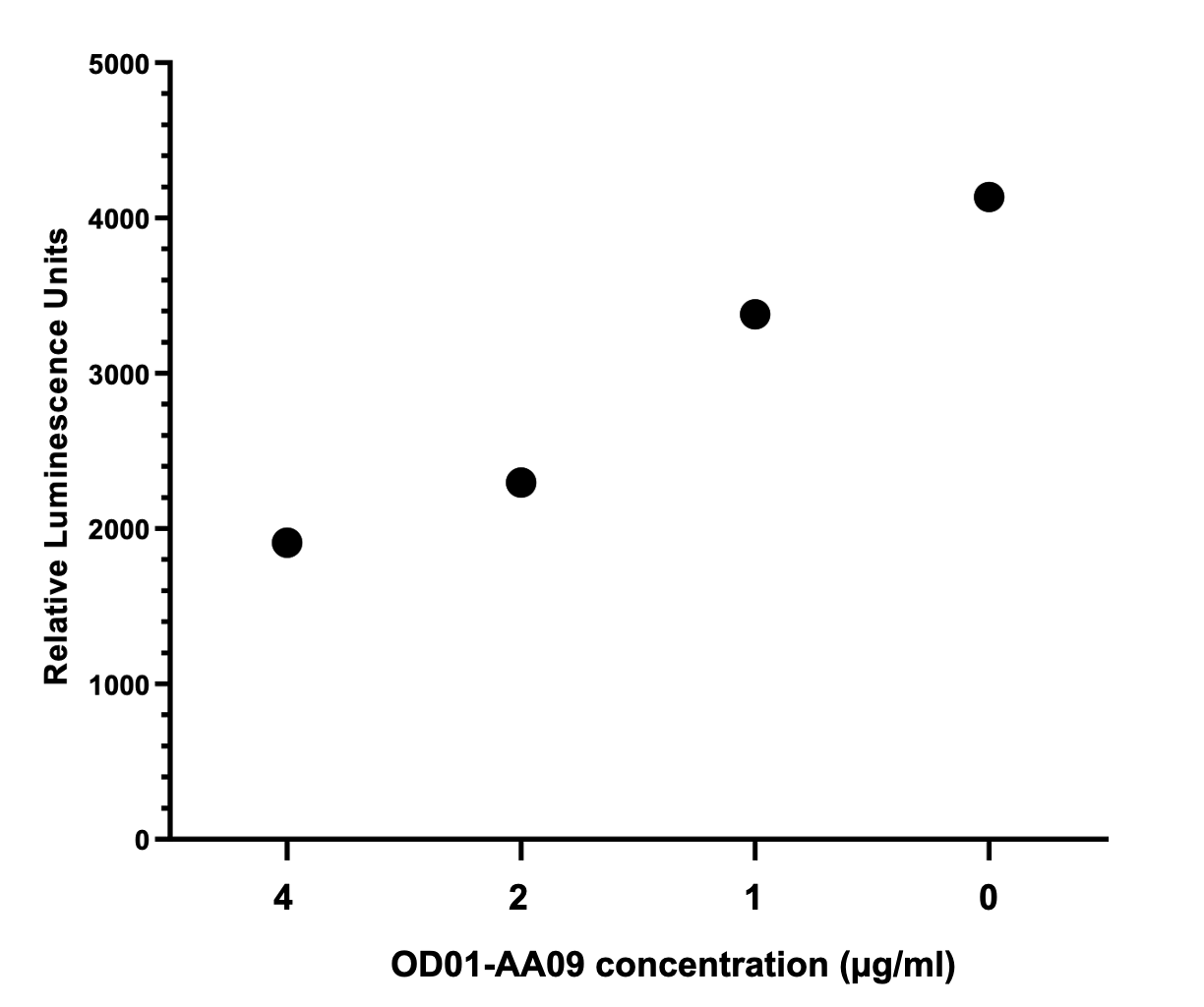

Supplement: FIG S8 [file msphere.00568-22-s0008.tif]
